# Online Support Groups for Family Caregivers: Scoping Review

**DOI:** 10.2196/46858

**Published:** 2023-12-13

**Authors:** Rosemary Daynes-Kearney, Stephen Gallagher

**Affiliations:** 1 Study of Anxiety, Stress and Health Laboratory Department of Psychology University of Limerick Limerick Ireland; 2 Health Research Institute University of Limerick Limerick Ireland

**Keywords:** caregivers, carer, caregiver, caregiving, informal care, family care, unpaid care, spousal care, carers, online support groups, scoping review, review methods, review methodology, social support, review, support, peer support, online support, development, communication, psychosocial, life experience, caregiver needs, engagement

## Abstract

**Background:**

Caregiving can affect people of all ages and can have significant negative health impacts on family caregivers themselves. Research has shown that social support acts as a buffer against many negative health impacts. A common source of social support is support groups. Although traditionally, these groups were conducted in a face-to-face setting, the advent of the internet, social media applications, and the smartphone have seen online support groups (OSGs) develop as a space where many caregivers seek support. The number of OSGs has increased exponentially, but there is no clear consensus on what factors or characteristics of OSGs contribute to social support development within them or what types of OSGs are available to family caregivers.

**Objective:**

This study aimed to conduct a scoping review to contribute to the understanding of the types and characteristics of OSGs for family caregivers.

**Methods:**

Following the Preferred Reporting Items for Systematic Reviews and Meta-Analyses Extension for Scoping Reviews guidelines, the CINAHL, PsychInfo, Psych Articles, Social Sciences, Communication Source, Medline, and Web of Science databases were searched for studies (caregiver focused, adults aged 18 years or older, online social support groups, caring for a living person, peer-reviewed journal publications on empirical research). In total, 19 studies were included in the review. The research questions were (1) what type of social support groups are online for adult family caregivers, (2) what the communication mediums and characteristics of these OSGs are, and (3) what psychosocial or other factors make OSGs successful or unsuccessful for participants.

**Results:**

In response to the first research question, we found that the majority of OSGs took place on public text-based forums and were illness specific. Where demographics were reported, participants were predominately women, White, and working with university-level education. There were a variety of caregiving relationships. For the second research question, the most common communication medium found was text-based communication, with the use of emojis, photos, and GIF (Graphics Interchange Format) files as part of these exchanges. Most frequently, the OSGs were asynchronous with a degree of anonymity, not time-limited by the frequency of contact or duration, and moderated by peer or professional moderators or facilitators. Results for the third research question explored the overarching categories of safe communication and engagement and group management. These described successful OSGs as having a focus on similar others with shared lived experiences communicated in a nonjudgmental space overseen by trained peer or professional facilitators.

**Conclusions:**

There are several key considerations for OSGs to be successful for family caregivers. A general recommendation for practitioners is to give importance to building active moderation and multifaceted structures of support to meet different levels of caregiver needs and the ability to engage.

## Introduction

Family caregivers provide care to family members, friends, or others because of physical, neurological, or mental ill health or disability; care needs related to old age [1]; or addiction. The World Health Organization (WHO) estimates that there are approximately 349 million care-dependent people worldwide, each of whom requires either family or professional caregiving [2]. Caregiving can affect people of all ages, with estimates suggesting that between 2% and 8% of children in industrialized nations will be caregivers [3]. Studies have shown that 1 in 5 adults in Ireland and the United States and 1 in 8 adults in the United Kingdom are family caregivers [4].

Although there are economic savings, estimated to be between €2.1 (US $1.3) billion and €10 (US $10.9) billion in Ireland alone [1], there is often a health cost to these caregivers. Research on caregiving has consistently highlighted the negative health effects of caregiving on caregivers. These negative health effects include impacts on mental and emotional well-being [5,6], social isolation [7], and physiological effects, such as immune and hormonal impairments [8] and lower life satisfaction in young caregivers [9]. Moreover, caregivers who were initially healthy were found to have a greater future risk of illness and disability 7 years later, and the risk was still evident even in those who stopped caring at baseline [10].

Despite this, research has also demonstrated that caregiving can be a positive experience [11] and that the view that caregiving in and of itself is inherently stressful is an “overly narrow, simplified, and limited view on these types of human relationships” [11]. There are myriad factors predictive of health in family caregivers, including care-related factors (eg, disability type, behavior problems, hours caring), caregiver personal experiences (eg, coping styles, depression), and sociodemographic factors (eg, age, gender, and race) [5]. One factor that has been found to be health protective for family caregivers is social support [12].

Social support, “those social interactions or relationships that provide individuals with actual assistance or that embed individuals within a social system believed to provide love, caring, or a sense of attachment to a valued social group or dyad” [13], has been consistently correlated with positive health outcomes [14]. A recent meta-analysis found social support to be associated with reduced caregiver burden [12]. Moreover, social support, whether perceived or actual, has many health benefits for caregivers, such as positive correlations with psychological resilience [7,15], mediating effects of internalized stigma and caregiving burden [16], and coping and family quality of life for caregivers of individuals with autism [17]. Given its health-protective role, one can see why translating social support for the purposes of interventions for family caregivers has been the focus of research [17]. In fact, caregiving can be considered an ongoing stressful life event, and social support in the form of support groups can have a particular role, function, and meaning for family caregivers.

Caregiver social support groups provide an opportunity to relieve the caregiving burden [18]. They are peer-led or professional-led groups that “provide a safe environment in which caregivers can share their experiences, exchange information and find emotional support from others who...have an understanding of what someone is going through” [19]. The traditional model of support service provision for family caregivers has been in a face-to-face setting, which can present a logistical challenge for family caregivers who, by nature of their role, are largely time restricted, as well as often being unable to leave their loved ones without respite care or a similar support service [20]. As a result, family caregivers have not always been able to avail of social support when they require it, and as such, there has been a significant increase in the use of online social support groups.

Online support groups (OSGs) are where people come together to share, seek advice, and socialize in a virtual space [21]. Over the past decade, the number of OSGs has increased exponentially [22]. The growth of OSGs has also accelerated because of the COVID-19 pandemic, which has mirrored the growth of the caregiver burden in family caregivers [23]. For example, during the COVID-19 lockdown in 2020, 93.7% of family caregivers used smartphones to access support, with nearly 50% of overall respondents using technology to stay connected with caregiver or patient support organizations [23]. A recent review of the National Carers Strategy in Ireland by family caregivers themselves showed that they want to see new and advanced possibilities for online support to be included in the next iteration of this policy [24].

Despite this growth, there is no consensus on what factors or characteristics of OSGs contribute to the development of social support [14,25]. For example, are people bonding, sharing information, providing informational or emotional support, or doing all of these, and what types of groups work best, such as peer-led, moderator-led, or unmoderated groups? In addition, little is known about why family caregivers join or stay in these groups. As one can see, more research, in particular a greater understanding of what the psychological mechanisms behind these OSGs are and how they influence social support and its health benefits, is required. Further, OSGs can be considered a form of e-support intervention [26], and understanding the mechanisms through which caregiver interventions may have beneficial effects on support recipients is an understudied area of family caregiving [27].

Similarly, little is known about the types of OSGs that are available to family caregivers: Are they illness specific or more general, and are caregivers communicating via a synchronous or an asynchronous format? In fact, “participation in OSG does not guarantee that users receive the support they need” [28], and there is a poor understanding about the underlying theory or other factors influencing why caregivers choose to use or reject support services, with studies finding a phenomenon of caregivers rejecting available and affordable support [29]. Thus, exploration into the specific ways that caregivers benefit from and engage with OSGs will contribute to a greater understanding of the field and may highlight some of the key psychosocial factors of how OSGs work. These can be used to inform and better design OSGs to meet family caregiver needs.

The purpose of this study was to conduct a scoping review and to contribute to the understanding of the types and characteristics of OSGs for family caregivers. As there is no consensus, clarity, or guidelines on what constitutes an OSG for family caregivers, a scoping review was deemed suitable for examining emerging evidence [30]. In addition, a scoping review methodology was chosen as this allowed for a broad mapping of the available research in relation to OSGs for family caregivers [31].

## Methods

### Research Questions

The main concepts in the review questions were identified using the population, concept, and context (PCC) framework [32]. For this review, the population was adult family caregivers (aged 18 years or older), the concept was social support groups, and the context was research on OSGs published since 2010.

The primary research question of the scoping review was, What type of social support groups are online for adult family caregivers? Two further research questions were developed to probe more about the context and understanding of OSGs: What are the communication mediums and characteristics of these OSGs? What psychosocial or other factors make OSGs successful or unsuccessful for participants?

The research questions and the PCC framework enabled the construction of clear eligibility criteria for the inclusion and exclusion of papers. The inclusion criteria were studies that were caregiver focused and that included adults (age≥18 years), online social support groups, caring for a living person, and peer-reviewed journal publications on empirical research. The exclusion criteria were studies that were patient or noncaregiver focused and included young caregivers (age<18 years), a training/therapy/intervention program or similar, a bereavement support group, gray literature, reviews, discussions, opinions, and reports.

### Search Design

The PRISMA-ScR (Preferred Reporting Items for Systematic Reviews and Meta-Analyses Extension for Scoping Reviews) guidelines [33] informed the process and reporting of this scoping review (see Multimedia Appendix 1). The search design was registered on the Open Science Framework [34]. The search was not limited to papers in English, and although the initial search did return several papers in languages other than English, upon review after English translation, they did not meet the inclusion criteria. Therefore, all included papers were written in English. If papers were not available in the institution library, requests for copies were made using the interlibrary loan system. Of all requests made, only 1 did not return a paper.

### Search Strategy

An iterative search strategy was used in consultation with a specialist librarian to develop comprehensive search strings, such as the following, with additional search strings included in Multimedia Appendix 2:

- TX “online support” OR TX “web support” OR TX virtual support groups OR TX (online support groups or online support group) OR TX (internet-based interventions or eHealth or web-based or electronic health intervention or internet-based therapy) OR TX telehealth OR TX “online support community” OR TX (social media or Facebook or Twitter or Instagram or Snapchat or Tumblr or social networking) OR TX (online forums or online discussions) OR TX online peer support groups OR TX “social network”
- TX family care* or TX (family caregivers or information caregivers or relatives or family) OR TX (spouse or partner or wife or wives or husband or couple or couples) OR spousal caregiver
- S1 AND S2
- Limiters: published date 20100101-20201231
- Expanders: Apply equivalent subjects.
- Search modes: Boolean/phrase

As the focus of the scoping review was OSGs, a retrospective date of 2010 was chosen, as this is when smartphones came into public use, making access to online services more accessible and widely available [35]. The following databases, accessed via EBSCO, were searched: CINAHL, PsychInfo, Psych Articles, Social Sciences, Communication Source, Medline, and Web of Science. Snowball referencing was also used on papers that passed the full screening. Additionally, a call for possible relevant papers was circulated on social media and through a mailing list specifically for research relating to family caregivers. These approaches are consistent with several recently published scoping reviews [36-38].

### Screening of Studies

**Figure 1:**
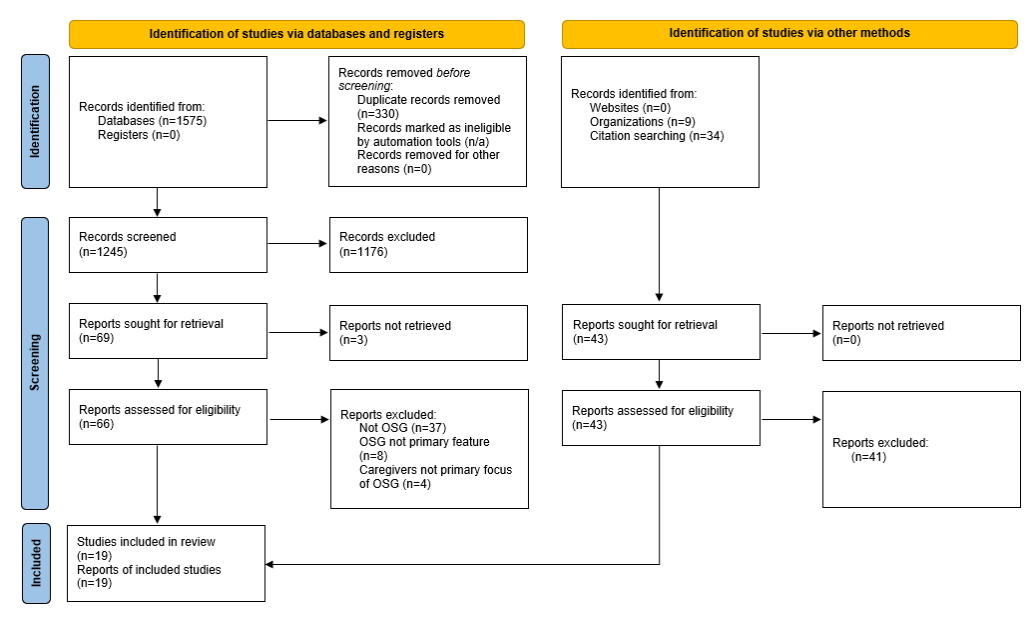
PRISMA flow diagram of the screening process. OSG: online support group; PRISMA-ScR: Preferred Reporting Items for Systematic Reviews and Meta-Analyses.

Title and abstract screening was guided by the inclusion criteria and completed by the first author. A simple abstract-screening tool was developed by the first author and a sample of 10 abstracts used to test the tool [39]. The tool was refined and agreed upon with the second author. The first author then completed the abstract screening. For resource management, the second author screened 10% of papers at this stage and all papers at the full-screening stage. Any disagreements on inclusion or exclusion were discussed and a decision reached by consensus. The PRISMA flow diagram [40] was used to record the screening process (Figure 1). Papers were exported to Endnote to check for duplicates. Exclusion of the remaining papers was due to 1 of the following reasons: not an OSG (another type of online intervention, typically psychoeducational), OSGs not the primary feature, and family caregivers not the primary focus of OSGs. At the end of the screening process, 19 papers were included for the review. Table 1 provides a summary of the papers included [41-59], with more in-depth information provided in Multimedia Appendix 3. The demographic characteristics of each study’s participants are summarized in Table S2 in Multimedia Appendix 3.

**Table 1:** Data extraction: papers included in the review (N=19).

Authors (year)	Title of paper	Type of family caregiver
Andersson et al [41]	The Experiences of Working Carers of Older People Regarding Access to a Web-Based Family Care Support Network Offered by a Municipality	Working caregivers of older people
Andréasson et al [42]	Developing a Carer Identity and Negotiating Everyday Life Through Social Networking Sites: An Explorative Study on Identity Constructions in an Online Swedish Carer Community	All adult family caregivers
Benson et al [43]^a^	Online Social Support Groups for Informal Caregivers of Hospice Patients With Cancer	Caregivers of hospice cancer patients
Clifford and Minnes [44]	Logging On: Evaluating an Online Support Group for Parents of Children With Autism Spectrum Disorders	Parents of children with ASD^b^
Cole et al [45]	Caregivers of School-Aged Children with Autism: Social Media as a Source of Support	Caregivers of children with ASD
Coulson and Greenwood [46]	Families Affected by Childhood Cancer: An Analysis of the Provision of Social Support Within Online Support Group	Families affected by childhood cancer
Diefenbeck et al [47]	Emergence of Yalom’s Therapeutic Factors in a Peer-Led, Asynchronous, Online Support Group for Family Caregivers	Caregivers of chronically ill individuals
Ferrell et al [48]	Informal Caregiving Experiences in Posttraumatic Stress Disorder: A Content Analysis of an Online Community	Caregivers of people with posttraumatic stress disorder (PTSD)
Friedman et al [49]^c^	Online Peer Support Groups for Family Caregivers: Are They Reaching the Caregivers With the Greatest Needs?	Caregivers of military personnel
Ihring et al [50]	Online Support Groups Offer Low Threshold Backing for Family and Friends of Patients With Prostate Cancer	Caregivers of people with prostate cancer
Knepper and Arrington [51]	Parents’ Narratives in an Online PHPV Forum: Toward a Typology of Caregiver Illness Narratives	Parents of children with persistent hyperplastic primary vitreous (PHPV)
Kruk [52]	‘I can’t bear the thought that he might not recognise me’: Personal Narratives as a Site of Identity Work in the Online Alzheimer’s Support Group	Caregivers of people with Alzheimer’s disease
Male et al [53]	The Continuous Confrontation of Caregiving as Described in Real-Time Online Group Chat	Caregivers of people with cancer
McKechnie et al [54]	The Effectiveness of an Internet Support Forum for Carers of People With Dementia: A Pre-Post Cohort Study	Caregivers of people with dementia
Mohd Roffeei et al [55]	Seeking Social Support on Facebook for Children With Autism Spectrum Disorders (ASDs)	Parents of children with ASDs
Oprescu et al [56]	Online Information Exchanges for Parents of Children With a Rare Health Condition: Key Findings From an Online Support Community	Parents of children with clubfoot
Trail et al [57]^c^	The Relationship Between Engagement in Online Support Groups and Social Isolation Among Military Caregivers: Longitudinal Questionnaire Study	Caregivers of military personnel
Washington et al [58]	Factors Influencing Engagement in an Online Support Group for Family Caregivers of Individuals With Advanced Cancer	Caregivers of people with cancer
Yoo et al [59]	Sociocultural Determinants of Negative Emotions Among Dementia Caregivers in the United States and in Korea: A Content Analysis of Online Support Groups	Caregivers of people with dementia

^a^This study used data from the same randomized pragmatic trial sponsored by the National Cancer Institute (R01CA203999).

^b^ASD: autism spectrum disorder.

^c^These studies used the same online support group (OSG) for part or all of their research sample: the Military Veteran Caregiver Network (MVCN).

### Data Analysis

The data analysis process incorporated frequency counts and inductive qualitative content analysis [32]. The 19 papers were imported into a shared project in NVivo version 1.7.1.1534 (QSR International), which was used for inductively coding papers against the research questions. NVivo was chosen as a tool to support the analytical process due to flexibility of the software features [60], such as memo writing, a search tool to highlight coding patterns, and its visualization options for presentation [32].

**Figure 2:**
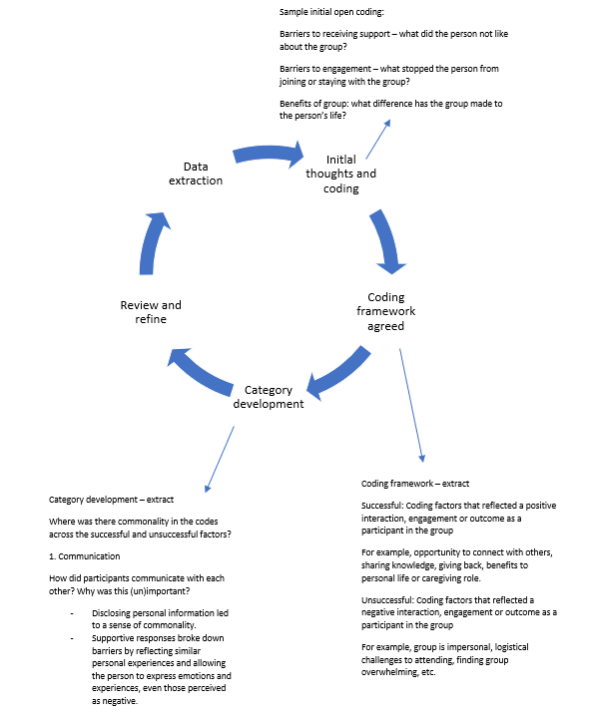
Iterative inductive data analysis process.

An inductive approach to extraction and analysis was chosen as there is no single agreed-upon framework for OSGs for family caregivers. The first author used a process of open coding in the first instance, which was refined to a coding framework agreed upon with the second author. A data extraction table was developed using this coding framework in Microsoft Excel to summarize key information for the first 2 research questions (What type of social support groups are online for adult family caregivers, and what are the communication mediums and characteristics of these OSGs?); see Tables S3 and S4 in Multimedia Appendix 3. For the third research question (What psychosocial or other factors make OSGs successful or unsuccessful for participants?), the analysis process was iterative, with repeated readings of the papers and ongoing team discussions to refine the codes into overarching categories (Figure 2) [4,60]. As with the screening process, any disagreements between the researchers were resolved through discussion and consensus.

## Results

### Types of OSGs Available Online for Adult Family Caregivers

Most OSGs took place on public text-based forums [7], a public social networking site (SNS) group [1], private text-based forums [5], closed web networks [2], private and secret SNS groups [3], closed private messaging groups [1], and private email forums [1]; see Table S4 in Multimedia Appendix 3. In addition, 14 (73.7%) OSGs were illness specific, 1 (5.3%) was for chronically ill people, 2 (10.5%) were for carers of older people, and 2 (10.5%) were for military caregivers. Of the 19 studies, 15 (78.9%) researched 1 (5.3%) OSG, Coulson and Greenwood [46] studied 3 (15.8%) OSGs, while Friedman et al [49], Mohd Roffeei et al [55], and Yoo et al [59] studied 2 (10.5%) OSGs.

Not all studies reported demographics of the research participants. Several studies included analyses of message postings, so descriptives, such as age and gender, could not be reported. Of studies that did report demographics, the participants were mostly women (female: n=536, 83.4%; male: n=107, 16.6%), White (n=391, 84.2%; Black/other: n=73, 16.8%), and working with university-level education. Group members on forums about illness that affected children mainly comprised parents. Other OSGs dealing with illnesses, such as dementia and cancer, had a variety of different relationships with the cared-for person, such as adult child, spouse, or another relative.

### Communication Mediums and Characteristics of OSGs

The communication mediums of the OSGs were extracted under 1 category, text/video, and the characteristics of the OSGs were divided into 4 categories: asynchronous/synchronous, type of moderation, anonymity level, time-limited interaction. These were deductive categories based on the previous knowledge and experience of the authors as practitioners, researchers, and family caregivers themselves. It was more difficult to extract these data from the text, as the information was not always clearly stated when the study described the OSGs being researched. In some cases, the information was not available. This was most noticeable in the moderation and anonymity level categories (see Table S4 in Multimedia Appendix 3).

In total, 13 of the OSGs operated solely in text-based mediums, while 6 provided text-based support with additional support options available, such as online educational resources. In addition, 17 of the OSGs were asynchronous, 1 was synchronous, and 1 asynchronous OSG offered an additional monthly videoconference session [57]. Group moderation involved peer-moderated, public-facing forums and a mix of peer and professional-led private groups on SNSs or dedicated web settings. OSGs on Facebook were different from public web forums, in that they “are intentionally planned, have a specific purpose, structure and rules and guiding principles” [58].

It appears that group membership was commonly as a registered user, either on an anonymous basis or a pseudonymous basis. In some instances, group membership could be registered through the caregiver’s known and verified social media account. Two of the OSGs were not anonymous, where part of the moderator’s role was to introduce new members when they joined the group. Only 1 OSG was clear that participants would be introduced personally, as their children all attended the same service [45]. Of the 19 studies, 6 (31.6%) were not clear about the level of anonymity in the OSGs. One OSG was time limited, the synchronous OSGs met for a set period each week, and the OSGs operated for a specified period (9 or 10 weeks) [53]. OSGs dealing with hospice or palliative care had a natural ending where members were removed following the death of their loved one.

In summary, the most common communication medium was text-based communication, with the use of emojis, photos, and Graphics Interchange Format (GIF)s as part of these exchanges. Most frequently, the OSGs were asynchronous, text-based groups not time limited by the frequency of contact or the duration of the group, had a degree of anonymity, and were moderated by peer or professional moderators or facilitators.

### Psychosocial or Other Factors of OSGs That Make Them Successful or Unsuccessful for Participants

All studies found that OSGs were beneficial to family caregivers and that even where OSGs had low-threshold requirements for entry, social support was built [52]. Informational and emotional support were extracted as the most common types of support prevalent in the OSGs. Across all studies, participants were generally satisfied with the support that they received and found the OSGs useful. Therefore, overall, the OSGs were successful in providing support to family caregivers involved in these studies.

Informational support was present as advice, referrals, situation appraisals, and education [46,55]. Emotional support was seen through expressions of affection, empathy, sympathy, and encouragement [46,55]. The motivations for joining an OSG were to exchange specific information, help others, and improve one’s own social well-being [50]. The OSGs also offered an opportunity for the caregivers to grow [53], and in some cases, participants felt that they had become better caregivers [53,54]. Washington et al [58] found that the context (emotional isolation and caregiving downtime), content (topics and notifications), and delivery method (private Facebook group) motivated engagement with the OSG, while Yoo et al [59] concluded that the level of negative emotions experienced by the caregivers influenced their seeking of emotional or other forms of support in OSGs.

Several of the studies provided insights into the needs of caregivers experiencing emotional turmoil, challenges in interpersonal relationships, and barriers to care [48]. Caregivers of those with neurological and psychological conditions were more likely to seek online support than other caregivers [49]. Although spouses of those with prostate cancer reported high levels of distress, the highest distress scores were in adult children of patients with prostate cancer, which may highlight an unrecognized group of caregivers [53]. Cultural factors, such as the relationship between caregiver and cared-for person [59] and the social acceptance of illness or disability [45], could influence whether a caregiver would seek to use an OSG and the types of messages that they may post [59].

**Figure 3:**
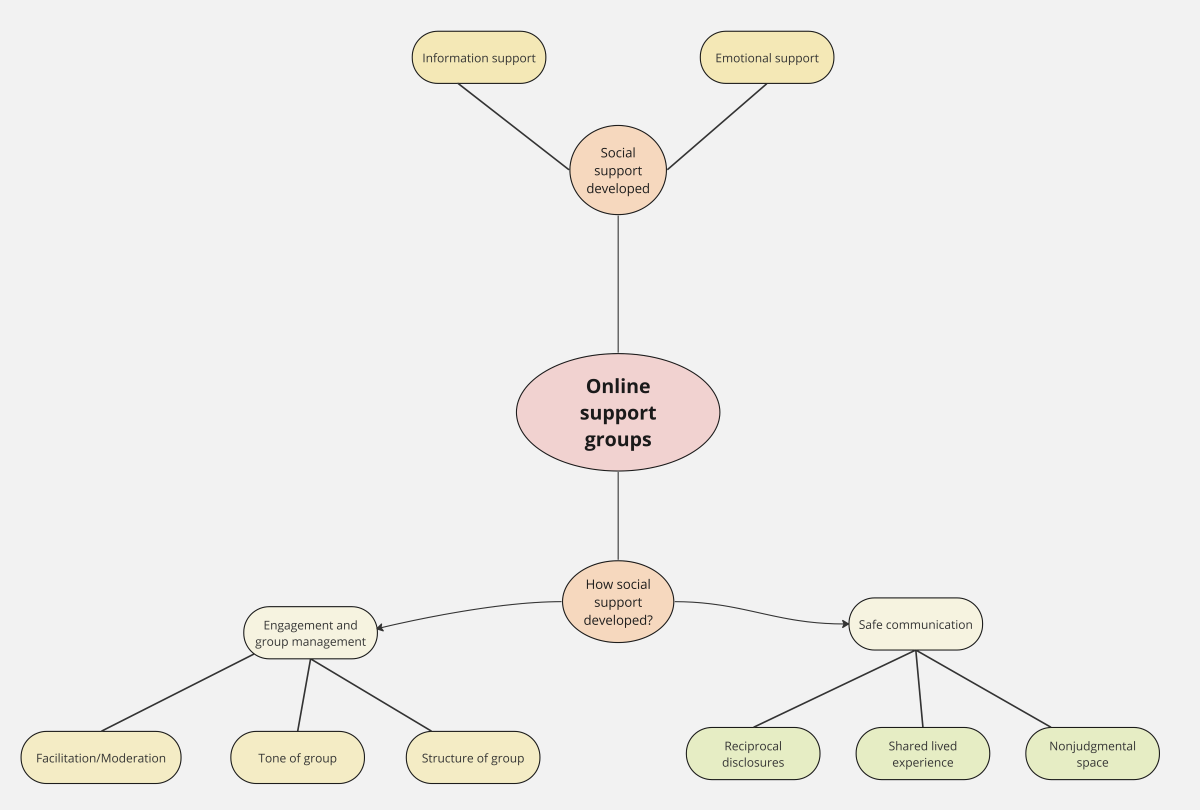
Illustration of categories of social support generated and how this social support was generated in successful OSGs. OSG: online support group.

To further understand the factors underlying the provision of social support in the OSGs, 2 overarching categories of factors were generated to explain what contributed to the success of the OSGs, with 3 subcategories in each (see Figure 3 and Table 2). The first category was “safe communication within the OSG,” with the subcategories “reciprocal disclosures,” “shared life experiences,” and “nonjudgmental space.” The second category was “engagement,” with the subcategories “tone of the group,” “facilitation/moderation,” and “structure of the group.”

**Table 2:** Key elements of categories.

Category and subcategories		Key elements
**Safe communication: The OSG^a^ needs to be experienced as safe and supportive to build social support**		
	Reciprocal disclosure	Self-disclosure of information and emotions Patterns of engagement for eliciting and providing support Giving support as helpful as receiving
	Shared life experiences	Personal narratives Second stories Emotional support in comments Use of nonverbal tools Exchange of learned knowledge and experience
	Nonjudgmental space	Expressions that would normally be stigmatized Second stories Exploration of identity Importance of similar others: experience, not demographics, key factor of similarity Importance of closeness of relationship with cared-for person
**Engagement and group management: These are conditions necessary to enable social support to be built and positive experiences of the OSG**		
	Facilitation/moderation	Use of relevant professionals beneficial Active role needed in developing group cohesion Create tone, culture, and safety in the group Encourage engagement: responding to posts Monitor content
	Tone of the group	Low engagement protective action against the negative tone of the group Low engagement protective action against the emotional impact of the group content
	Structure of the group	Flexibility main benefit of online format OSG relieves social isolation Asynchronous format encouraged engagement Real or perceived anonymity encouraged disclosures Privacy extremely important No clear definition of engagement

^a^OSG: online support group.

### Safe Communication Within the OSG

From the included studies, it was evident that for the OSGs to be successful in building social support, they needed to be experienced as a safe and supportive space. This finding was regardless of the theoretical or empirical approach of the study (see Table S1 in Multimedia Appendix 3). As seen before, a main characteristic of OSGs is that they are text based, and several of the included studies examined the content of the messages themselves, with 1 (5.3%) study focusing on only sent, not received messages [56] and the remaining 18 (94.7%) considering 2-way exchanges.

#### Reciprocal Disclosures

Self-disclosure, both emotional and informational, was a common method of interacting in the OSGs [43]. Emotional self-disclosure often accompanied other statements, such as patient updates, or appeared in comments [55], while informational self-disclosures were what new members first posted. Direct requests for social support were uncommon and, where they did occur, were requests for informational support [43]. Information seeking may also have been accompanied by self-disclosure as a method to obtain responses from the community and generate trust [56].

Where the OSGs were targeted at specific cohorts of caregivers, the information support was specific to the condition (eg, for parents of children with autism spectrum disorder [ASD], communication support was a key area [45]). The culture of the caregiver may influence their likelihood of posting emotional messages; for example, Korean caregivers were more likely to post emotionally charged messages compared to US caregivers [59].

There were patterns of engagement in both eliciting and providing support, with the ideal structure of posts for eliciting support combining verbal self-disclosures with nonverbal emotional self-disclosures, such as emojis or GIFs [43]. Initiating messages included a description of the cared-for person’s diagnosis with a question for others [51]. The first messages of participants took the format of a welcome, often with detailed information in response to the question, and encouraging statements and messages of empathy and prayers [43,51,55]. The participant messages involved the “phenomenon of second stories,” highlighting the commonality of shared experience, with providing support being as helpful as receiving support [45].

#### Shared Life Experiences

The OSGs functioned with an emphasis on the life experience of the group members [47], and in the written forums, participants used personal narratives when communicating [52]. Telling personal stories helps people to “voice and give order to disconnected life experiences...as well as achieve a sense of cohesion...to articulate grievances...and promot[e] caregiver-led local re-definitions of certain morally contested aspects of...identity” [52]. Similarly, social recognition in OSGs may be an important way of buffering against negative experiences in the caregivers’ lives [42].

This sharing of second stories was important for “fostering social relationships and connectedness online” [52]. A high percentage (73%) of messages on a forum were intended for an individual recipient [56], with emotional support being conveyed within comments or responses to posts rather than first postings [55]. Where nonverbal tools, such as GIFs, emojis, and images, were used in addition to text content, they elicited more support than posts without nonverbal content [43]. These patterns of communication served to build network support, with some members organizing to meet at offline events [55].

The studies showed that caregiver identity and well-being were developed through the exchange of learned knowledge and experience, with 60% of sources of information in a single OSG coming from personal experience [56], and these authors suggested that it was this similar experience rather than demographics, such as age and gender, that was most important to fostering social support. Although professionals and family caregivers may have frequent interactions in relation to the cared-for person, some caregivers felt their expertise was dismissed by “professionals” [42], with their knowledge seldom being considered. Being able to share their expertise with others and be socially recognized as a valuable repository of knowledge came through in many studies as a way for the caregiver to feel seen and empowered and to feel that they had something to offer [41,42,54].

#### Nonjudgmental Space

Across all studies, posts where disclosures that would normally be stigmatized in offline settings, such as expressions of anger or negative emotions at or toward the cared-for person, were sensitively responded to with reciprocal narratives of group members’ own experiences. Caregivers used the OSGs to express anxious or worried thoughts about guilt, self-blame, compassion fatigue, sexual disruption, and maltreatment by the cared-for person [48]. The second stories were important for dealing with expressed negative emotions, where support developed through themes of universality and by offering hope [47].

The OSGs were found to be safe spaces where family caregivers could explore their own identity and how this was affected by providing care. Many participants of the different OSGs expressed how their own identities were dominated by the caring role and that the OSGs were a forum where “an invisible self can become visible” [42]. This was evident by participants making a distinction between themselves as a carer and as a person, using the space to state their own needs and as a safe space where they could express things that they would not say to family members for fear of negative reactions.

The OSGs provided changes in the identity work of family caregivers in 2 key areas: (1) disrupted family relationships or significant other relationships signifying “a new us” [53] and (2) normalization of atypical patterns of behavior [52]. Although for parents, there may be less disrupted identity, as the role of the parent is naturally in a caregiving role, “there may be additional pressures due to the diagnosis...but in the end members are parents” [51], for caregivers in other family relationships, caregiving can disrupt not just their lives but also their sense of self when “navigating disequilibrium” [53]. It was important that the OSGs be made up of similar others [54] as “collective connectedness” with a group of people who share similar experiences or identity was found to lead to a decrease in social isolation and foster psychological well-being [57].

In summary, OSGs were successful in generating social support, where participants felt safe to express their life experience, including nonsocially acceptable disclosures, without judgment. Two-way exchanges of information and emotional support enabled members to feel valued as a person and a valid source of knowledge. OSGs operated as a forum to process a range of complex emotions, relationships, and experiences, where statements of universality and hope were 2 key features of the OSGs.

### Engagement and Group Management

Although the first overarching category considers why the OSGs were successful, the second overarching category of engagement and group management draws together factors that indicate the conditions necessary to allow these factors to develop. Caregiving was reported as being a significant life interference for the caregiver [48], such as excess responsibilities, limited or no respite from the person or caring situation, forced adaptation of life to fit the needs of the cared-for person [42], and having to cope without adequate support from others [53]. This life interference could be seen to have an impact on how often and how long a person engaged with an OSG, where those with additional informal caregivers were most likely to use the OSGs they were members of [49]. Some participants, even when they had other sources of social support, still felt alone prior to joining an OSG [50].

Engagement in OSGs may be affected by the relationship to the person being cared for, with spouses participating in OSGs early in the course of cancer [50]. The benefits of membership in OSGs were greater for those who engaged the most [51]. The relationship between engagement and social isolation was mediated by increases in participant interactions [57], where engagement was associated with a decrease in social isolation at 6 months. There was a general consensus across the studies for the need to develop and offer appropriate OSGs early in the caregiving process that adapt across the course of the caring lifespan [41], as different types of support forums with different levels of engagement may be needed to derive benefits [54].

Despite these positive associations, engagement was not defined and explored in all studies. In 1 (5.3%) study, engagement was measured by the frequency of visits to OSGs, as well as the time spent during visits [49]. Another study defined passive users as those who logged in and took part in a discussion (the most frequent form of engagement) as opposed to those who actively posted on the forum [42]. Lurking, defined as not posting in the OSG, made up 33% of participants in 1 (5.3%) study, with the remaining group members spending more than 10 hours in the OSG [50].

Interestingly, participants expressed that they did not need to post to benefit—that “it’s more useful for me to read other people’s experiences” [43]. Similarly, attendance on the OSG cannot be considered synonymous with receiving a benefit from the OSG [58]. This indicates that our understanding of engagement in OSGs needs to be examined to investigate what users consider engagement and how it impacts them.

#### Facilitation/Moderation

Across the 19 studies, effective moderation was found to encourage engagement. There was a recognized need for the moderators and facilitators to set the parameters, rules, and culture of the OSG to ensure that it met the stated objectives. Many of the OSGs in the studies used relevant professionals as facilitators, with 2 (10.5%) studies proposing nurses as ideal candidates to facilitate OSGs [43,47]. Participants found having the presence of health care professionals a valuable resource, both for creating a feeling of safety in the OSG and for validating information that other members of the OSG provided [44]. Where OSGs were peer-led only, there was a desire to have more educational or informational content from relevant professionals in the field [42,43]. However, participants in both peer-led and professional-led OSGs reported many of the same benefits.

Moderation took on different forms, such as “monitoring and supporting” [41], playing an active role in setting topics for discussion, redirecting conversation, keeping conversation flowing, opening and closing sessions [42], and quality-checking the advice and information shared on the OSG [45]. Facilitators played a role in creating the culture, tone, and feeling of safety of the OSGs. The facilitators and moderators seemed to have an active role in developing group cohesion, which, without active action, may not develop or develop to a lesser extent in a nonmoderated OSG [47]. Some participants were not sure as to what was expected of them in the OSGs or what the objective of the OSGs was, which could lead to them posting about irrelevant topics [45]. Active engagement, such as replying to posts, may have been affected by the role of the moderator, with participants assuming that the moderator would respond to posts [57]. Similar to the issue with engagement, none of the included studies provided a clear definition of facilitator and moderator, which this paper recommends for future research to understand whether there is a difference in their roles and functions.

Additional frustrations that could reduce engagement are a lack of replies to posts [46] and inappropriate or judgmental posts or comments [54]. Although these frustrations were more evident on large public-facing forums, participants generally reported that the volunteer moderators on these sites were helpful. In OSGs that were designed with designated moderators, the moderators enabled engagement through responding to posts and encouraging others to do the same, as well as monitoring the content for inappropriate material [54]. These actions seem to have been instrumental in creating safety in the OSG. The monitoring of posts pre- and postpublication safeguarded against inappropriate posts and inaccurate information. The purpose of the OSG could be reinforced and the tone of the OSG influenced, created, and maintained by interactions of the facilitators, moderators, and the OSG.

There were statements of universality and hope found across the 19 studies, which are 2 key features of Yalom’s 11 therapeutic factors of group development and address how change occurs in support groups. One measure of success is the “extent to which it cultivates therapeutic group factors” [47]. Although information sharing was found to be critical to the cohesiveness of an OSG, “certain therapeutic factors...do not naturally emerge without active cultivation by professionally trained group leaders” [47]. It was necessary to “maximize factors that promote meaningful member engagement, responding to changes in activity and tone over time” [58].

#### Tone of the Group

“Users appear motivated to engage with personally relevant, trustworthy content that is delivered in a positive tone via an easy-to-use platform” [58]. Low levels of engagement in the OSGs were described across many studies as a protective mechanism against a negative tone, negative discussions, or a feeling of being overwhelmed by the stories of others in the group. This was particularly noticeable when posts dealt directly or indirectly with the death of the cared-for person. Members of a hospice OSG described the “strain they experienced by being exposed to others’ grief,” which, due to the context of the OSG (run in a hospice during end-of-life care), happened on a frequent basis [42]. Paradoxically, keeping the tone upbeat and light also prevented some participants from contributing to the OSGs as they were worried about bringing the other participants down [45].

#### Structure of the Group

A number of studies considered whether online support without face-to-face groups could contribute to or sustain social isolation. Interestingly, the opposite was found across all the 19 studies, as the flexibility of the online support space (as demonstrated in the aforementioned characteristics) was 1 of the main benefits expressed by participants. The relief from social isolation was prevalent regardless of the medium of the OSG, with the OSGs generally being described as user friendly and easy to use. New technologies could serve as a barrier to engagement [41]. To address this, several OSGs provided instructions on how to use both the technology and the group itself and had people on hand to provide help if this was required by the participants. This was found to be helpful and overcame a barrier to engagement for members who would have dropped off due to fear or lack of experience with new technologies [41].

The asynchronous nature of the OSGs was reported as a facilitator to engaging with support compared to face-to-face or synchronous groups, which require more time and effort. However, the absence of the “here and now” interaction in asynchronous groups could affect the development of group factors in an online setting [47]. Some members of an asynchronous OSG did express a desire to have a face-to-face group, although this appeared to be a desire for a live chat rather than to be in the same place as other people [43]. Although real-time synchronous online groups more closely approximate a face-to-face in-person group, a synchronous online group results in inconsistent attendance at the scheduled times, with scheduling conflicts being the most common reason reported for missing a group interaction [44].

For forum groups, anonymity was seen as an important factor, providing the freedom to “vent sensitive or embarrassing topics” [59] and creating a “disinhibition effect,” with higher levels of personal information shared via the computer compared to face to face [47] and with a “reduction in social cues [members are]...less inhibited to discuss socially delicate and embarrassing topics” [52] and a “safe place to be open with those dreaded feelings [I did] not want to have” [53].

A key consideration about OSGs is whether there is true anonymity in Facebook groups and many public forums. Privacy and group confidentiality were factors in choosing the secret Facebook group (now called private Facebook group) format of OSGs. Facebook has a requirement that one’s verified name be used on its platform. Although Facebook does offer the option to post anonymously, this is only if the group admins and anonymous posts are available to the user and the group. Additionally, the username and profile picture are still visible to the group’s admins and moderators, as well as to Facebook.

Similarly, many public forums have the author’s name displayed, whether the true name or a pseudonym, and posts may reveal details that would enable the poster to be identified [54], including details about the cared-for person [55]. Another risk of text-based OSGs is that there is a written record of what is shared. Even in secret Facebook groups, content can be copied from the group and posted on participants’ personal walls [57]. However, this real or perceived anonymity of OSGs may account for the high levels of self-disclosure seen across all the OSGs [46].

In summary, engagement is a necessary factor for OSGs to be successful for participants; however, a clear definition of what is meant by engagement in OSGs needs to be developed. Engagement can be supported by clear, active, consistent facilitation and moderation to monitor and respond to the content, culture, tone, and levels of engagement in the OSG. Engagement may be affected by cultural factors, the relationship between the caregiver and the cared-for person, and where the person is in their caregiving journey.

## Discussion

### Principal Findings

This review aimed to gain a better understanding of the types of available OSGs and the characteristics of OSGs for adult family caregivers, including the psychosocial factors underlying their successful provision of social support. Overall, the review found that OSGs are beneficial to participants as social support is generated and they provide a place to connect with others in similar situations and relieve some negative impacts of caregiving. Research on face-to-face caregiver support groups has found similar benefits, such as improving the quality of life [61] and sense of togetherness [62].

Although there is variety in the type of mediums of OSGs, most OSGs in this review were illness specific, comprising caregivers who had different relationships with the cared-for persons. The research groups predominately comprised White, middle-aged women. Although on the one hand, this could be considered a WEIRD (White, educated, industrialized, rich, and democratic) bias in the research [63], especially as few of the 19 studies explicitly noted the racial or ethnic make-up of the groups, on the other hand this reflects the global reality that most caregiving is performed by women aged between 50 and 65 years [64].

The analysis found that the asynchronous and perceived anonymity of the online space are key characteristics that support engagement due to its flexibility. The text-based nature of the OSGs, another key characteristic, demonstrates an inbuilt bias in the research, assuming that participants are digitally literate with access to the internet and devices. This bias also presents an opportunity to explore barriers to family caregivers not engaging in OSGs, a cohort that none of the studies reached.

The overarching categories of “safe communication” and “engagement and group management” indicate factors that can elucidate why and how OSGs are successful. Categorization of these factors is important to address gaps in the existing knowledge and literature and for practitioners to understand when establishing and running OSGs. Each study concluded that there is a role of OSGs as a cost-effective, responsive, and efficient means of supporting family caregivers. There is a risk of investing in and establishing online groups without a thorough understanding of how and why they work and do not work for participants. For this reason, this paper summarizes the recommendations made in the studies (see Table S6 in Multimedia Appendix 3).

Our findings on safe communication and engagement are consistent with contemporary literature. A recent systematic review found that parents/carers of children with long-term physical conditions connect online with other parents in similar conditions for comfort and to reduce loneliness with the use of shared experiences reinforcing trust and validating their experiences [65]. Additionally, a mix of professional and peer support was found to be most effective in improving caregivers’ psychological well-being [66]. Several of the categorizations of empowering (exchanging information, sharing experiences, and finding recognition and understanding) and disempowering (inappropriate behavior online, information overload, and misinformation) processes in OSGs were also seen in these studies [67].

Of note in this review is the assertion across all studies of the value of the OSG comprising similar others, which supports the recent finding that “people with similar experiences can give others hope, inspiration and guidance in ways that are different from professionals” [68]. Identification is a key element of social support, and OSGs are a valid social categorization for people’s social identity [28], with group cohesion dependent on categorization and identification [69]. Collective identity groups [70] were found to have a stronger significant mediation between online participation and perceived social support [28]. Recent research on social identity in OSGs for family caregivers found that group identity can be cultivated through considered, active, and balanced moderation [71] and is part of the growing body of literature called the “social cure” using social identity frameworks to better understand the benefits of social groups [72].

There is a consensus across research that OSGs are used by vulnerable cohorts of caregivers, who have the fewest resources provided to support their caregiving role. Although many of the studies encouraged professionals to refer family caregivers to OSGs, they also highlighted that OSGs may not be appropriate for everyone. OSGs should not be confused with other types of online interventions, as the objective of OSGs is to provide support rather than support behavior change [73]. OSGs may not be the most appropriate support for caregivers with psychological distress or for those who wish for specific training. Therefore, professionals need to be clear as to why they are referring the family caregiver to an OSG and whether it is appropriate to the caregiver’s needs.

Similarly, not all OSGs consider the gender, racial, cultural, or faith-based background of participants, which may impact the efficacy of the support [74]. Motivations for caregiving can be influenced by cultural underpinnings, such as filial piety, familism, and repayment and reciprocity [75], and support services should be sensitive of specific cultural values [75]. Recent research on a culturally specific face-to-face OSG helped caregivers manage and rethink their caring processes and values [76]. The studies in this review concluded that paying due attention to what is being raised in OSGs may provide indications for what type of appropriate interventions can be developed or run to support the members [53,54]. Further research into the effectiveness of different types of OSGs through a systematic review and an exploration of culturally tailored groups would be beneficial.

Across the 19 studies, the OSGs were used as a source of information gathering, with users’ personal experiences valued highly as sources of information. Although hospitals and health care providers may be considered a source of truth for medical information, the reliance on person-to-person information indicates that there may be an information gap in traditional medical providers’ sources of communication with family caregivers. The quality of information received either online or from physicians can mitigate caregiver uncertainty [77], and this indicates the importance of ensuring they have readily available and easily understood up-to-date information that is communicated across online platforms.

Many of the studies did not clearly state the level of anonymity and moderation in the OSGs. Privacy was one of the highest determinants that facilitated or impeded implementation of eHealth groups, with “anxiety often felt by participants about using technology to document personal issues” [78]. Future research should clearly include the characteristics of OSGs to facilitate an understanding of how these may have influenced findings and how these can be applied to similar groups. Privacy in OSGs needs to be further explored to address any barriers that levels of anonymity may present to participants.

Similarly, there was no consistent definition of engagement across the studies. In general, engagement as a concept is ill-defined and has underlying assumptions that it is inherently effective with positive outcomes [73]. Engagement in online spaces is complex, with participants often making “in the moment” decisions about whether to engage, high levels of attrition, and coexisting states of positive and negative engagement, with the latter not always being ineffective [73]. Further work would be beneficial to develop a consistent understanding of effective engagement in OSGs.

Active facilitation and moderation were seen across all studies as an important component in supporting engagement and developing group cohesion. Notably, the role of the moderator/facilitator differed depending on the format of the OSG. In several of the studies, the facilitators played a dual role as participant and group leader. Although the role appears to have worked in these studies, it raises questions about what support is available for facilitators in their own caregiving role and about the sustainability of the model, with the risk of facilitators leaving or of being harmed. In practical terms, this indicates that it is necessary to build robust structures with multiple facilitators and provide support and supervision care for staff to ensure that the OSG service is maintained. More understanding about the relationship between facilitators/moderators and engagement in OSGs is important.

This scoping review indicated many areas that would benefit from further research, including researching how social inequality and access to digital resources may impact family caregivers accessing OSGs and how different types of family caregivers or different lengths of time as a family caregiver may lead to different experiences of online support and cultural elements relating to engagement and the use of OSGs.

### Limitations

There is an inherent bias in the studies as all participants were, by default, literate and had access to the internet and the research dealt primarily with text-based expressions. As such, it must be acknowledged and is likely that the studies excluded caregivers who did not meet these requirements. There was also a wide range of theoretical frameworks and tools used, which limits the standardization of findings. There was no consistency in reporting on some key terms that we common across all OSGs, such as the type, key characteristics, and the meaning of engagement in the group.

Many studies relied on self-selection of participants. This may indicate that those participating in the studies were more motivated and likely to engage in support both on- and offline, and this, again, missed caregivers who fell outside this bracket. As such, the findings in the selected studies and in this review itself may not be generalizable or transferable to all family caregivers.

There is a general issue of bias in research toward White caregiver groups in the United States [74]. Although a strength of this review is that it captured studies from different countries and cultures, the ethnic make-up of samples from outside the United States was not always recorded. Where the participant group was ethnically diverse, a positive outcome of the OSG was greater awareness and knowledge of cultural differences [45]. This paper advocates that regardless of where the research is conducted, it is important to specify, where possible, the racial or ethnic make-up of the participants to be transparent about the production of WEIRD research. This paper also advocates for more research on caregivers from other cultural groups.

Many studies had solely or predominately participants who were women and low variability in different ethnic and socioeconomic backgrounds, where reported. OSGs have been proposed as a tool to provide support for hard-to-reach groups of caregivers, such as caregivers who are men [79] and minority groups [74]. The review shows that research into OSGs has not yet captured these cohorts and suggests wider systemic issues that need investigation.

None of the studies provided an analysis of racial and gendered roles/relationships between moderators and group engagement. Although worldwide, family caregivers are predominately women, there are also a large number of family caregivers who are men. Where reported, the facilitator were also women. This may have limited the interactions and engagement of caregivers who are men in the OSGs. Similarly, perceived social support, a measure used across many of the reviewed studies, is reflected differently across race and gender [80], reinforcing the need for greater clarity on participant groups and concepts in this area.

Finally, the 2 authors did not screen all papers, which may have introduced bias or led to papers being missed; however, the recommended 10% minimum criterion was applied [39]. The authors also used the full body of the text to extrapolate data, and it is recognized that errors may have occurred on the part of the authors.

### Conclusion

This review shows that there are several key factors to be considered for an OSG to be successful for family caregivers. The 2 overarching categories of safe communication and engagement describe OSGs with a focus on similar others and shared life experiences communicated in a nonjudgmental space overseen by trained peer or professional facilitators. Moreover, these categories also fit with a social identity framework, a framework being applied successfully to understand the health benefits of social groupings [70]. Although there are several limitations to the studies, a general recommendation for practitioners is that it appears important to build in active moderation and multifaceted structures of support to meet different levels of caregiver needs and the ability to engage. This paper suggests that the findings of this scoping review indicate a systematic review would be beneficial to further explore key categories highlighted in this review.

## Appendix

Multimedia Appendix 1 PRISMA-ScR (Preferred Reporting Items for Systematic Reviews and Meta-Analyses Extension for Scoping Reviews) checklist.

Multimedia Appendix 2 Search strings.

Multimedia Appendix 3 Data extraction tables for each research question. Information includes demographic details, types of groups, membership characteristics, communication mediums, and summary of recommendations.

## Abbreviations

ASD: autism spectrum disorder
GIF: Graphics Interchange Format
OSG: online support group
PCC: population, concept, and context
PRISMA-ScR: Preferred Reporting Items for Systematic Reviews and Meta-Analyses Extension for Scoping Reviews
SNS: social networking site

## Data Availability

All relevant data are available withing the manuscript.
