# Supplementary material for: Online Support Groups for Family Caregivers: Scoping Review
Source: J Med Internet Res. 2023 Dec 13;25:e46858. doi: 10.2196/46858 (PMC10753418; doi:10.2196/46858)
Supplement: Multimedia Appendix 2 [file jmir_v25i1e46858_app2.pdf]

Additional search strings

A:

1. TX family care*
2. TI online support groups or online support group
3. S1 AND S2
4. TX "online support" OR TX "web support" OR TX virtual support groups OR TX (online support groups or online support group) OR TX (internet-based interventions or ehealth or web-based or electronic health intervention or internet based therapy) OR TX telehealth OR TX "online support community" OR TX (social media or facebook or twitter or instagram or snapchat or tumblr or social networking) OR TX (online forums or online discussions) OR TX online peer support groups OR TX "social network ...
5. TX family care* OR TX (family caregivers or informal caregivers or relatives or family) OR TX (spouse or partner or wife or wives or husband or couple or couples) OR TX spousal caregiver
6. S4 AND S5
7. (S4 AND S5) NOT (bereavement or grief or loss or mourning or death) NOT (drugs or alcohol or substance abuse) NOT parenting NOT (parents or mother or father or parent) NOT (eating disorders or anorexia or bulimia or disordered eating or binge eating disorder) NOT (education or school or learning or teaching or classroom or education system)
8. (S4 AND S5) NOT TX (bereavement or grief or loss or mourning or death) NOT TX (drugs or alcohol or substance abuse) NOT TX parenting NOT TX (parents or mother or father or parent) NOT TX (eating disorders or anorexia or bulimia or disordered eating or binge eating disorder) NOT TX (education or school or learning or teaching or classroom or education system)
9. TI (S4 AND S5) NOT TI (bereavement or grief or loss or mourning or death) NOT TI (drugs or alcohol or substance abuse) NOT TI parenting NOT TI (parents or mother or father or parent

) NOT TI (eating disorders or anorexia or bulimia or disordered eating or binge eating disorder) NOT TI (education or school or learning or teaching or classroom or education system)

10. TI family care* OR TI (family caregivers or informal caregivers or relatives or family)
11. S4 AND S10
12. TI (S49 AND S55) NOT TX (bereavement or grief or loss or mourning or death) NOT TX (drug addiction or drug abuse or substance abuse) NOT TX (eating disorders or anorexia or bulimia or disordered eating or binge eating disorder) NOT TX (education or school or learning or teaching or classroom or education system)

B:

1. TX “online support groups”
2. TX “family caregivers or “family carers”
3. S1 AND S2
4. NOT ABSTRACT “young carers” or “young caregivers”
5. NOT TITLE “patients with”

Limiters – Published Date: 20100101-20201231

Expanders – Apply equivalent subjects.

Search Modes – Boolean/Phrase

C:

1. TI (caregiv* or family) OR AB (caregiv* or family)

   AND
2. TX online or internet or web or social media

   AND

3. TX support

   AND

4. TX group* (group, groups).

D:

1. TX family care*

2. TI online support groups or online support group

3. S1 AND S2

6. TX family care* OR TX (family caregivers or informal caregivers or relatives or family) OR TX (spouse or partner or wife or wives or husband or couple or couples) OR TX spousal caregiver

7. TI DE "Caregivers" OR "Caregiving"

8. (DE "Online Social Networks" OR DE "Internet Usage" OR DE "Social Media" OR DE "Social Networks" OR DE "Computer Mediated Communication" OR DE "Online Community") OR (DE "Online Social Networks")

9. TI DE "Support Groups" OR DE "Social Networks" OR DE "Social Support"

10. (DE "Internet") AND (DE "Electronic Health Services" OR DE "Digital Interventions" OR DE "Internet")

11. TI DE "Group Identity" OR DE "Social Identity"

12. S7 AND S9

Abstract Screening Tool

1. Does this paper involve empirical research?
2. Has it been published in a peer-reviewed journal?
3. Does this paper involve research about adult family caregivers?
4. Are family caregivers the primary focus of the research?
5. Are caregivers caring for a living person?
6. Does this paper involve research about online support groups?
7. Is the online support group a social support group or another form of group?
