# Supplementary material for: Online Support Groups for Family Caregivers: Scoping Review
Source: J Med Internet Res. 2023 Dec 13;25:e46858. doi: 10.2196/46858 (PMC10753418; doi:10.2196/46858)
Supplement: Multimedia Appendix 3 [file jmir_v25i1e46858_app3.pdf]

Table S1

Data Extraction Table: Articles included in review.

Paper	Title	Year	Location	Research Methodology	Theoretical Approach	Types of Family Caregiver
Andersson, S., Erlingsson, C., Magnusson, L., Hanson, E.	The experiences of working carers of older people regarding access to a web-based family care support network offered by a municipality	2017	Sweden	Qualitative	None specified	Working caregivers of older people
Andréasson, F., Andreasson, J. & Hanson, E.	Developing a carer identity and negotiating everyday life through social networking sites: an explorative study on identity constructions in an online Swedish carer community	2017	Sweden	Qualitative	Constructionist approach	All adult family caregivers
Benson, J., Parker Oliver,D., Washington, K.T., Rolbeicki, A., Lombardo, C.B., Garza., J.E., Demiris, G.+	Online social support groups for informal caregivers of hospice patients with cancer	2020	USA	Mixed methods with concurrent nested design	Berbee et al (1993) Social support activation model	Caregivers of hospice cancer patients
Clifford, T., & Minnes, P.	Logging On: Evaluating an online support group for parents of children with ASD	2012	USA	Quantitative	Stress buffering model (Cohen & Wills, 1985)	Parents of children with Autism Spectrum Disorder
Cole, L., Kharwa, Y., Khumalo, N., Reinke, J. & Karrim, S.	Caregivers of School-aged Children with Autism: Social Media as a Source of Support	2017	South Africa	Qualitative	Not stated	Caregivers of children with Autism Spectrum Disorder

Coulson, N.S. & Greenwood, N.	Families affected by childhood cancer: an analysis of the provision of social support within online support group	2012	UK and USA	Qualitative	Cutrona & Suhr Social support scale (1992)	Families affected by childhood cancer
Diefenbeck, C.A., Klemm, P.R., Hayes E.R.	Emergence of Yalom's Therapeutic Factors in a Peer-Led, Asynchronous, Online Support Group for Family Caregivers	2014	USA	Qualitative	Yalom's Therapeutic Factors	Caregivers of chronically ill individuals
Ferrell, E.L., Russin, S.E., & Hardy, R.M.	Informal caregiving experiences in posttraumatic stress disorder: A content analysis of an online community	2018	USA	Qualitative	Transactional stress theory; Caregiver stress theory	Caregivers of people with PTSD
Friedman, E., Trail, T., Vaughan, C.A. & Tanielian, T.*	Online peer support groups for family caregivers: are they reaching the caregivers with the greatest needs?	2018	USA	Quantitative	None stated	Caregivers of military personnel
Ihring, A., Renner, T., Muck, T., Maatz, P., Borkowetz, A., Keck, B., Maatouk., Wirth, M. P., Huber, J.	Online support groups offer low-threshold backing for family and friends of patients with prostate cancer	2018	Germany	Quantitative	None stated	Caregivers of people with prostate cancer
Knepper, K. & and Arrington, M.	Parents' Narratives in an Online PHPV Forum: Toward a Typology of Caregiver Illness Narratives	2016	USA	Qualitative	Frank's Typology of Illness narratives	Parents of children with Persistent Hyperplastic Primary Vitreous

Kruk, B.	‘I can’t bear the thought that he might not recognise me’: Personal narratives as a site of identity work in the online Alzheimer’s support group	2015	UK	Qualitative	Conversation analysis and membership categorization analysis	Caregivers of people with Alzheimer’s Disease
Male, D.A., Fergus, K.D., Stephen, J.E.	The Continuous Confrontation of Caregiving as Described in Real-Time Online Group Chat	2015	Canada	Qualitative	Grounded theory	Caregivers of people with cancer
McKechnie, V., Barker, C., & Stott, J.	The Effectiveness of an Internet Support Forum for Carers of People With Dementia: A Pre-Post Cohort Study	2014	UK	Mixed methods	None stated	Caregivers of people with dementia
Mohd Roffeei, S. H. M., Abdullah, N., & Basar, S. K. R.	Seeking social support on Facebook for children with Autism Spectrum Disorders (ASDs)	2015	Malaysia	Qualitative	Social support behaviour code	Parents of children with Autism Spectrum Disorders
Oprescu, F., Campo, S., Lowe, J., Andsager, J., Morcuende, J.A,	Online Information Exchanges for Parents of Children With a Rare Health Condition: Key Findings From an Online Support Community	2013	Not specified	Qualitative	Uncertainty Management Theory and Brashers information seeking behaviour scheme	Parents of children with clubfoot
Trail, T., Friendman, E., Rutter, C., & Tanielian, T.*	The Relationship Between Engagement in Online Support Groups and Social Isolation Among Military Caregivers: Longitudinal Questionnaire Study	2020	USA	Quantitative	None stated	Caregivers of military personnel

Washington, K., Parker Oliver, D., Benson, J., Rolbiecki, A., + Jorgensen, L., Cruz-Oliver, D., & Demiris, G. +	Factors influencing engagement in an online support group for family caregivers of individuals with advanced cancer	2018	USA	Secondary Qualitative Data Analysis	Context, content, delivery framework (Perski et al, 2014)	Caregivers of people with cancer
Yoo, J. H., Jang, S., & Choi, T.	Sociocultural Determinants of Negative Emotions Among Dementia Caregivers in the United States and in Korea: A Content Analysis of Online Support Groups	2010	Korea and USA	Mixed methods	Uses and gratification model of communication media (Weiser, 2001)	Caregivers of people with dementia

Note:

+ These studies used the data from the same randomized pragmatic trial sponsored by the National Cancer Institute (R01CA203999).

* These studies used the same OSG for some or all of their research sample: Military Veteran Caregiver Network (MVCN).

Table S2

Demographic details extracted from included papers.

Paper	Number of Participants	Age of Participants	Gender	Ethnicity	Relationship to Cared-For Person[1]	Employment and Education[2]
Andersson et al (2017)	N = 9	Age range = 34-64 years	F = 9	Not reported	Adult child = 7<br>Spouse = 1<br>Other = 4	8 full-time employed;<br>2 university-level education
Andréasson et al (2017)	N = 44	Age range not reported.<br>Mean age = 64.7 years	F = 34<br>M = 10	Not reported	Spouse = 30<br>Parent = 11<br>Other = 3	Mainly retired with higher-level education
Benson et al (2020) +	N = 58	Age range not reported.<br>Mean age = 56.54 years	F = 47<br>M = 11	Black/African American = 7<br>White/Caucasian = 49<br>Other = 2	Spouse = 13<br>Parent = 7<br>Adult child = 29<br>Other = 9	21 with household income over $70,000;<br>23 with university-level education
Clifford & Minnes (2012)	N = 20	Age range = 33 to 53 years.<br>Mean age = 43 years	F = 20	Not provided	Parent = 20	Median income = €83,000;<br>18 with university-level education
Cole et al (2017)	N = 6	Age range = 23-48<br>(5 participants only provided age).	F = 5<br>M = 1	Black = 3<br>Indian = 3	Parents = 6	No reported
Coulson & Greenwood (2012)	Analysis of messages on online group	N/A	N/A	N/A	N/A	N/A
Diefenbeck et al (2014)	N = 11	Age range = 35-62.<br>Mean age = 49.36	F = 10<br>M = 1	White/Caucasian = 11	Spouse = 3<br>Adult child = 5<br>Parent = 2<br>Other = 1	All employed

[1] In some studies, participants were caring for more than one person, and so counts do not add up to sample size.
[2] Different studies reported different measures. Here we have summarized highest or median level of income, highest level of education or if caregivers were working.

Ferrell et al (2018)	Analysis of messages on online group	N/A	N/A	N/A	N/A	N/A
Friedman et al (2018)*	N = 242	Not reported	Not reported	Not reported	Not reported	96% employed
Ihring et al (2018)	N = 83	Mean age = 53.7 years	F = 47<br>M = 36	Not reported	Spouse = 27<br>Adult child = 26<br>Other = 30	51 higher-level education (A-levels)
Knepper & Arrington (2016)	Analysis of messages on online group	N/A	N/A	N/A	N/A	N/A
Kruk (2015)	Analysis of messages on online group	N/A	N/A	N/A	N/A	N/A
Male et al (2015)	N = 25	Age range = 27-75 years.<br>Mean age = 51 years	F = 19<br>M = 6	Not reported	Spouse or partner = 19<br>Adult child = 5<br>Parent = 1	Not reported
McKechnie et al (2014)	N = 119	Age range = 22-86 years.<br>Mean age = 56 years	F = 99<br>M = 18<br>Missing = 2	White British = 112<br>White other = 4<br>Other = 3	Adult child = 67<br>Partner = 38<br>Other = 20	58 employed;<br>42 retired;<br>57 university- or higher-level education
Mohd Roffeei et al (2015)	Analysis of messages on online group	N/A	N/A	N/A	N/A	N/A
Oprescu et al (2013)	Analysis of messages on online group	N/A	N/A	N/A	N/A	N/A
Trail et al (2020)*	N = 212	Age under 40 years = 124<br>Age 40-59 years = 74<br>Not provided = 14	F = 199<br>M = 13	Non-Hispanic white = 166<br>Hispanic = 23<br>Other = 23	Spouse or partner = 189<br>Other = 23	Not reported
Washington et al (2018) +	N = 58	Age range not reported.<br>Mean age = 56.54 years	F = 47<br>M = 11	Black/African American = 7<br>White/Caucasian = 49<br>Other = 2	Spouse = 13<br>Parent = 7<br>Adult child = 29<br>Other = 9	21 with household income over $70,000;<br>23 with university-level

						education
Yoo et al (2010)	Analysis of messages on online group	N/A	N/A	N/A	N/A	N/A

Note:
+ These studies used the data from the same randomized pragmatic trial sponsored by the National Cancer Institute (R01CA203999).
* These studies used the same OSG for some or all of their research sample: Military Veteran Caregiver Network (MVCN).

Table S3
Data Extraction Table – Question 1: Types of online support groups and membership characteristics.

Paper	Type of OSG	Illness-Specific OSG	Relationship to Cared-For Person	Type of Social Support
Andersson et al (2017)	Closed web network	No, caregivers of older people	Mixed	Informational; emotional
Andréasson et al (2017)	Private text-based forum	No, caregivers of older people	Mixed	Informational; emotional
Benson et al (2020)	Private SNS group	Yes	Mixed	Emotional
Clifford & Minnes (2012)	Private text-based forum	Yes	Mixed	Social connection; informational support
Cole et al (2017)	Messaging app	Yes	Mixed	Skill acquisition; emotional support; informational support
Coulson & Greenwood (2012)	Public text-based forum	Yes	Mixed	Emotional and informational
Diefenbeck et al (2014)	Closed web network	Yes	Mixed	9 therapeutic factors present: Top 3 are Group Cohesiveness, Catharsis, and Imparting Information
Ferrell et al (2018)	Public text-based forum	Yes	Mixed	Emotional support; informational support
Friedman et al (2018)	Private text-based forum	No, caregivers of military personnel.	Mixed	Seeking relief from caregiver burden – higher level of burden more likely to engage

Ihring et al (2018)	Public text-based forum	Yes	Mixed	Informational support, relief from feelings of uncertainty
Knepper & Arrington (2016)	Email forum	Yes	Parents	Informational support; emotional support
Kruk (2015)	Public text-based forum	Yes	Mixed	Emotional support; informational support
Male et al (2015)	Private text-based forum	Yes	Mixed	Breaking social isolation; emotional support
McKechnie et al (2014)	Public text-based forum	Yes	Mixed	Social similarity; informational support; emotional support
Mohd Roffeei et al (2015)	Public SNS Group	Yes	Parents	Informational support; emotional support; network support; esteem
Oprescu et al (2013)	Public text-based forum	Yes	Parents	Informational support; emotional support
Trail et al (2020)	Private text-based forum	No, caregivers of military personnel	Mixed	Breaking social isolation
Washington et al (2018)	Private SNS group	Yes	Mixed	Informational or educational support
Yoo et al (2010)	Public text-based forum	Yes	Mixed	Korean caregivers seeking emotional support; US caregivers seeking informational support

Table S4

Data Extraction Table – Question 2: What are the communication mediums and characteristics of these online support groups (OSG)?

Paper	Type of Communication	Type of Moderation	Primary Mode of Communication	Anonymity Level	Group Duration
Andersson et al (2017)	Asynchronous	Professional, Trained	Text based	Registered users	No limit
Andréasson et al (2017)	Asynchronous	Professional, Trained	Text based	Not stated	Unclear – seems ongoing
Benson et al (2020)	Asynchronous	Professional, Trained	Text based	Registered Facebook account	Unclear – person would be removed from group following brief transition period once bereaved
Clifford & Minnes (2012)	Synchronous	Professional, Trained	Text based	Pseudonymous	
Cole et al (2017)	Asynchronous	Professional, Trained	Text based	None – participants known to each other in group	No limit – research participants had used group for 6 months or longer
Coulson & Greenwood (2012)	Asynchronous	Not specified	Text based	Not stated - Public forum	No limit
Diefenbeck et al (2014)	Asynchronous	Peer, Trained	Text based	Pseudonymous	16 weeks
Ferrell et al (2018)	Asynchronous	Mix of peer and professional	Text based	Not stated – public website with global reach	No limit
Friedman et al (2018)	Asynchronous	Mix of peer and professional	Text based	Registered users	Had joined group 6 months previously – no limit on duration of group membership
Ihring et al (2018)	Asynchronous	Professional, Trained	Text based	Registered users	No limit

Knepper & Arrington (2016)	Asynchronous	Peer	Text based	Anonymous	No limit
Kruk (2015)	Asynchronous	Peer	Text based	Not stated – public website	No limit
Male et al (2015)	Synchronous	Professional, Trained	Video	Registered users	9 or 10 weeks
McKechnie et al (2014)	Asynchronous	Peer	Text-based	Registered users	No limit – research participants had joined the group 12 weeks prior.
Mohd Roffeei et al (2015)	Asynchronous	Peer	Text based	Registered Facebook account	No limit
Oprescu et al (2013)	Asynchronous	Peer	Text based	Not stated – public website with global membership	No limit
Trail et al (2020)	Asynchronous	Professional, Trained	Text based	Registered users	Had joined group 6 months previously – no limit on duration of group membership
Washington et al (2018)	Asynchronous	Professional, Trained	Text based	Registered Facebook account	Unclear – person would be removed from group following brief transition period once bereaved
Yoo et al (2010)	Asynchronous	Not specified	Text based	Not stated – public Alzheimer forum	No limit

Table S5

Key elements of categories

Category Name	Key Elements
**Category 1: Safe Communication**	The group needs to be experienced as safe and supportive to build social support.
Sub-Category	
Reciprocal Disclosure	• Self-disclosure of information and emotions<br>• Patterns of engagement for eliciting and giving support<br>• Giving support as helpful as receiving
Shared Lived Experience	• Personal narratives<br>• Second stories<br>• Emotional support in comments<br>• Use of non-verbal tools<br>• Exchange of learnt knowledge and experience
Non-Judgmental Space	• Expressions that would normally be stigmatized<br>• Second stories<br>• Exploration of identity<br>• Importance of similar others–experience, not demographics, key factor of similarity<br>• Importance of closeness of relationship with cared for person
**Category 2: Engagement and Group Management**	Conditions necessary to enable social support to be built and positive experiences of group.
Sub-Categories	
Facilitation/Moderation	• Use of relevant professionals beneficial<br>• Active role needed in developing group cohesion<br>• Create tone, culture, and safety in group<br>• Encourage engagement–responding to posts<br>• Monitor content
Tone of Group	• Low engagement protective action against negative tone of group<br>• Low engagement protective action against emotional impact of group content
Structure of Group	• Flexibility main benefit of online format<br>• OSG relieves social isolation<br>• Asynchronous format encouraged engagement

- Real or perceived anonymity encouraged disclosures
- Privacy extremely important
- No clear definition of engagement

Table S6

Summary of recommendations from the studies.

Paper	Recommendations: Actions to be Taken
Andersson et al (2017)	1) Web-based interventions must be offered early in the caregiving process.<br>2) Support functions need to be adaptable over the course of the caring trajectory.<br>3) Support must be offered in a way that will provide most assistance for the support to be experienced as meaningful.
Andréasson et al (2017)	4) Include concepts of caregiver identity.<br>5) Recognize caregiver knowledge, capacity, and life situation.<br>6) Cultural change within health and social care systems and practices to recognize and support carer contributions.
Benson et al (2020)	7) Use monitoring feature on Facebook to pre-approve content for accuracy and appropriateness before publication.<br>8) Strategies needed to strengthen relationships and increase interactions among group members – develop companionship support.
Clifford & Minnes (2012)*	9) Ensure facilitator is experienced and comfortable working with group.<br>10) Ensure able to think quickly to intervene during sessions.<br>11) Clearly outline format and expectations at beginning of group.<br>12) Find ways to encourage regular attendance.<br>13) Encourage participants to take a role in deciding focus and direction of the group.<br>14) Encourage development of relationships among participants.
Cole et al (2017)	15) Group needs rules, parameters, and defined roles to keep group focused on objective of group.
Coulson & Greenwood (2012)	16) Need mechanism to review actions of moderators to ensure they are appropriately meeting duties (eg, facilitating exchange of personal details between members).
Diefenbeck et al (2014)	17) Facilitator may need to be trained in group therapy.<br>18) Facilitator may need to emphasize member-to-member interactions.<br>19) Strategies need to be taken to support group cohesion.<br>20) Consider how OSG can be developed to meet heterogenous groups (demographically, caregiving intensity, relationship to cared-for person etc.).
Ferrell et al (2018)	21) Design materials based on caregiver needs.<br>22) Services that provide supports for patients (PTSD in this case) should

	consider incorporating a peer support group for caregivers.
Friedman et al (2018)	23) Target group to meet needs of the profiles of caregivers most likely to use the service.<br>24) Tailor system to support adaptive processes, such as interruptions and multi-tasking due to caregiving demands.
Ihring et al (2018)	25) Physicians should be aware of OSG.
Male et al (2015)	26) Develop counselling program or workshops to improve communication between caregivers and their loved ones navigating change in circumstances.
Oprescu et al (2013)	27) Tailor messaging for audiences, targeted messaging
Washington et al (2018)*	28) Designers of OSG tools should address the context of use and the needs and expectations of the target audience in system design.<br>29) Consider how appropriate new tool is to family caregivers–should not require extensive training and should be flexible–usability considerations when selecting and implementing OSG systems.<br>30) Healthcare teams offering OSG for family caregivers should ensure that group facilitators regularly monitor group activity, striving to balance the sharing and validation of difficult life experiences with content that is inspirational and uplifting.<br>31) Capitalize on existing behaviour patterns and offer OSG via popular social media platforms–may be able to serve more caregivers–need to address privacy and security concerns.<br>32) Effective support group facilitation requires an approach that is responsive to ever-changing group composition and dynamics.
Yoo et al (2010)	33) Practitioners, when they communicate with caregivers, should be extensively familiar with their characteristics, especially in terms of the primary negative emotions that they are experiencing.

Note:

* These articles had dedicated sections of recommendations for practitioners. For the remainder of the articles, information was extracted from the discussion and conclusion sections.
